# Which personality traits are necessary conditions for problematic alcohol use? Insights from a 23‐year longitudinal study

**DOI:** 10.1111/add.70417

**Published:** 2026-04-07

**Authors:** Angela Giugovaz, Peter Prinzie, Miranda C. Lutz, Ingmar H. A. Franken, Igor Marchetti

**Affiliations:** ^1^ Department of Life Sciences University of Trieste Trieste Italy; ^2^ Faculty of Psychology and Educational Sciences, Department of Developmental, Personality and Social Psychology Ghent University Ghent Belgium; ^3^ Center for Substance use and Addiction Research, Department of Psychology, Education and Child Studies Erasmus University Rotterdam Rotterdam the Netherlands; ^4^ Department of Health Sciences University of Florence Florence Italy

**Keywords:** conscientiousness, longitudinal, necessary causality, necessary condition analysis, personality traits, prevention, problematic alcohol use

## Abstract

**Background and aims:**

Personality traits have been consistently linked to alcohol use. High neuroticism and extraversion and low agreeableness and conscientiousness are known risk factors for both alcohol use frequency and problematic use across the lifespan. These associations have mostly been studied in the sufficient causality domain (“if X, then probably Y”), whereas little is known about these relationships in the necessary causality one (“if not X, then not Y”). Knowing that a variable is a necessary cause for an outcome helps identify who will be at risk for the outcome and who will be virtually immune. The aim of this study was to test whether personality traits in childhood, adolescence and emerging adulthood might serve as necessary conditions for problematic alcohol use in adulthood.

**Design:**

The study is part of the “Flemish Study on Parenting, Personality, and Development”, and it is a 23‐year longitudinal study across four time points [childhood (T1), adolescence (T2), emerging adulthood (T3), adulthood (T4)].

**Setting:**

Flanders (Belgium).

**Participants:**

At T1, the total sample consisted of 306 participants (age = 7.8 ± 1.13 years, and 59.15% females), at T2 of 289 participants (age = 15.78 ± 1.16 years, 59.86% females), at T3 of 290 participants (age = 21.78 ± 1.15 years, 59.66% females) and at T4 of 306 participants (age = 30.08 ± 1.13 years, 59.15% females).

**Measurements:**

Personality traits were assessed in childhood, adolescence and emerging adulthood using the Hierarchical Personality Inventory for Children (HiPIC) and were related to problematic alcohol use, measured through the Alcohol Use Disorders Identification Test (AUDIT), in adulthood. Necessary Condition Analysis (NCA) was used to test whether specific traits were necessary for the outcome, and to what extent.

**Findings:**

Conscientiousness emerged as a developmentally stable necessary condition for problematic alcohol use in adulthood (childhood: *d* = 0.31, *P* = 0.050; adolescence: *d* = 0.33, *P* = 0.052; emerging adulthood: *d* = 0.33, *P* = 0.023; all large effects). No other personality traits reached statistical significance.

**Conclusions:**

It is possible to identify, already in childhood and through the lifespan, personality characteristics that distinguish individuals vulnerable to developing problematic alcohol use in adulthood from those who are not. Specifically, lower levels of conscientiousness appear to be necessary to be at risk of potentially developing problematic alcohol use in adulthood, whereas high levels of conscientiousness appear to lead to virtual immunity.

## INTRODUCTION

Alcohol is the most commonly used psychoactive and addictive substance world‐wide. According to the World Health Organization (WHO) [1], Europe has the highest per‐capita consumption, with 62.4% of adults currently drinking. Although many consume alcohol occasionally, a notable proportion develops problematic use, which can lead to alcohol use disorder [2]. In Belgium, 7% of the adult population met the criteria for problematic use in 2018, defined as at least two indicators of alcohol‐related problems [3], including perceived loss of control, alcohol‐related guilt, social criticism and morning drinking. The rate typically rises to 9% in emerging to middle adulthood [4].

Problematic alcohol use is a multi‐dimensional construct that encompasses hazardous and harmful patterns of drinking as well as alcohol addiction symptomatology. It captures not only the frequency and quantity of consumption, but also the self‐reported negative physical, psychological and social consequences associated with alcohol use [5]. Importantly, it is not a diagnostic category in itself, but rather a broader dimensional construct that spans both subclinical and clinical levels of alcohol involvement [5]. Its development is shaped by individual and contextual risk factors from childhood through young adulthood [6, 7, 8].

Personality traits are key individual factors linked to both alcohol use frequency and problematic use across the lifespan. Recent evidence from a systematic review and meta‐analysis [9], which included 64 predominantly cross‐sectional studies, mostly conducted with samples of emerging adults, and adults confirmed these associations. Problematic alcohol use was consistently associated with low conscientiousness (e.g. being negligent, lazy, disorganized and quitting) [10] and low agreeableness (e.g. being suspicious, antagonistic, critical and irritable) [11], highlighting the persistent relevance of these personality dimensions. Longitudinal studies suggest that these associations may emerge early in life and develop through distinct pathways. In early childhood, temperamental traits such as sociability and early emotional or conduct difficulties are linked to later alcohol problems through specific personality dimensions [12]. Sociability predicts increased alcohol risk via higher extraversion (e.g. being talkative, affectionate, fun loving and active) [11] and sensation seeking (e.g. seeking novel and intense experiences, willing to take risks) [13]. Emotional and conduct difficulties predict risk through lower conscientiousness and emotional stability (e.g. feeling vulnerable and self‐conscious, experiencing negative emotions and worrying) [11, 12]. In adolescence, lower agreeableness and higher extraversion predict adult alcohol use [14]. In emerging adulthood, higher sensation seeking and impulsivity predict increased risk for later alcohol problems [15]. Findings regarding openness to experience have been less consistent. Several studies failed to find significant relationships with alcohol use, although lower openness has been associated with decreased alcohol consumption in some cases [9, 16].

Existing knowledge about the relationship between personality traits and problematic alcohol use has primarily been developed using traditional statistical methods, such as multiple regression and structural equation modeling (SEM). These models posit that higher levels of statistically significant predictors are associated with an increased probability of the outcome (e.g. lower levels of conscientiousness predicting higher probability of problematic drinking) [9]. Conceptually, these approaches follow probabilistic sufficiency logic (‘if X, then probably Y’) [17]. Whereas this framework has been extensively applied in psychological and medical research and has generated substantial insights, and it exhibits certain limitations. First, no predictor—regardless of strength—can guarantee an outcome, underscoring the probabilistic nature of such relationships. Consequently, findings provide information at the group level (average effect) and cannot be generalized to individual cases [17]. Second, the absence (or near‐0 magnitude) of a given predictor does not preclude the outcome, as other predictors may still lead to its manifestation (i.e. ‘if not X_1_, but X_2_, then probably Y’) [17]. For instance, individuals high in agreeableness, but low in conscientiousness may nonetheless develop problematic alcohol use [9].

Causality, however, is not limited to sufficiency, it also encompasses necessity [18]. The necessary causality framework focuses on identifying conditions that must be present for the outcome to occur, and without which it is virtually impossible [19]. Therefore, a factor is defined as a deterministic necessary condition if the outcome cannot happen in its absence (i.e. ‘if not X, then always not Y’). This perspective shifts the emphasis from estimating the probability of an outcome (i.e. probabilistic) to determining which conditions are essential for it to take place (i.e. deterministic) [17]. If a condition is necessary, then all cases in which the outcome is present must also show the presence of that condition and, in turn, all the cases without the necessary condition will not show the outcome. It follows that, pending multiple replications, a necessary condition identified on group samples can offer insights directly generalizable to the individual level (i.e. individualized care) [20]. Furthermore, a necessary condition cannot be replaced by another one. All the necessary conditions are required for the outcome to take place, and failing to meet one of those guarantees virtual ‘immunity’ from the negative outcome [21]. For instance, oxygen is a necessary condition for human life, and without it, life is impossible, even if other conditions like water are present.

Necessary condition analysis (NCA) [19] provides a formalized methodology for proposing and testing necessary conditions. NCA quantifies the extent to which a variable may function as a necessary condition for an outcome by providing both effect size and statistical significance. Recently introduced to clinical psychology and psychiatry by Marchetti and colleagues [21], NCA has shown promise in mental health research. For instance, rumination, depressive symptoms and stressful life events were necessary for the onset of at least one major depressive episode (MDE) within a 2‐year period among adolescents [22]. A key implication is that adolescents who did not reach the threshold levels of these three necessary factors were effectively protected from developing an MDE during the following 24 months. In contrast, adolescents who did meet these conditions were at risk—but not guaranteed—to experience the disorder. Similarly, intolerance of uncertainty has been identified as a necessary condition for the development of subclinical anxiety symptoms after 6 months in adolescents [23], and preliminary evidence showed that low self‐esteem is a necessary condition for developing symptoms related to anorexia, but not to bulimia, in adolescent girls after 12 months [24].

In the context of personality and problematic alcohol use, using necessary condition framework involves examining whether personality traits serve as deterministic necessary conditions for the emergence of this maladaptive behavior. This approach could identify threshold levels of traits that must be present for problematic use to occur—a question that, to our knowledge, has not yet been investigated, despite its potential to significantly deepen our understanding of maladaptive alcohol use.

In summary, this study aims to determine whether the Big Five personality traits—longitudinally measured from childhood to emerging adulthood—constitute necessary conditions for the later development of problematic alcohol use. Given the lack of specific theoretical frameworks identifying necessary conditions, our investigation is guided by current understanding rooted in sufficiency‐based models [19]. Hence, we hypothesize that low levels of conscientiousness and agreeableness during childhood (T1), adolescence (T2) and emerging adulthood (T3) are necessary conditions for high levels of problematic alcohol use in adulthood (T4; i.e. ‘if low levels of X, then maybe Y’ and ‘if high levels of X, then not Y’), and that high levels of neuroticism, extraversion and openness during childhood (T1), adolescence (T2) and emerging adulthood (T3) are necessary conditions for high levels of problematic alcohol use in adulthood (T4; i.e. ‘if high levels of X, then maybe Y’ and ‘if low levels of X, then not Y’).

## METHODS

### Sample

This study is based on the ongoing longitudinal Flemish Study on Parenting, Personality and Development (FSPPD) [25], initiated in 1999 and continuing to the present. Initially, a sample of elementary schools in Flanders (Belgium) was randomly selected, and within each school, children were selected based on birth date: names of children who had their birthday before 31 March were arranged in alphabetical order, and the second and the second‐to‐last child were chosen, ensuring random selection. Strata were then constructed based on three indices of representativeness, namely geographic location (province), sex and age. Families were contacted through the schools, and consent from both parents was obtained for participation. The study initially included four cohorts of elementary school‐age children (4–7 years old) [25, 26]. All participants are Flemish‐speaking Belgian citizens who have been followed‐up approximately every 3 years over 11 waves [25]. For the present study, four different waves were considered, namely wave 3 [(T1), 2001, 6–9 years old], wave 6 [(T2), 2009, 14–17 years old], wave 8 [(T3), 2015, 20–23 years old] and wave 11 [(T4), 2024, 29–32 years old], for a total of 23 years. Each one of these waves encompasses a distinct life stage, namely childhood, adolescence, emerging adulthood and adulthood. At T1, the total sample consisted of 306 participants (age = 7.8 ± 1.13 years, and 59.15% females), at T2 of 289 participants (age = 15.78 ± 1.16 years, 59.86% females), at T3 of 290 participants (age = 21.78 ± 1.15 years, 59.66% females) and at T4 of 306 participants (age = 30.08 ± 1.13 years, 59.15% females) (Table 1).

**TABLE 1:** Socio‐demographic characteristics.

	T1 (*n* = 306)	T2 (*n* = 289)	T3 (*n* = 290)	T4 (*n* = 306)
Age, years (SD)	6.3 (0.6)	15.8 (1.2)	21.78 (1.15)	30.08 (1.13)
Gender				
Male	41%	40%	40%	41%
Female	59%	60%	60%	59%
Mother's education				
Primary/secondary	7.84%			
Higher secondary education	50.33%			
Higher non‐university education	27.78%			
University education	13.40%			
No answer	0.98%			
Father's education				
Primary/secondary	12.42%			
Higher secondary	35.95%			
Higher non‐university	30.39%			
University education	19.28%			
No answer	2.29%			
Mother's job				
Not currently employed	14.71%			
Manual worker/blue collar	9.15%			
Clerical/employee	61.11%			
Self‐employed/managerial	13.07%			
No answer	2.29%			
Father's job				
Not currently employed	1.96%			
Manual worker/blue collar	20.59%			
Clerical/employee	40.20%			
Self‐employed/managerial	34.64%			
No answer	2.61%			
Educational track				
General secondary		73.01%		
Technical secondary		19.72%		
Artistic secondary		1.04%		
Vocational secondary		4.84%		
Special secondary		0.35%		
No answer		1.04%		
Highest degree				
No diploma/lower secondary education				12.42%
Higher secondary/vocational education				42.48%
University degree				39.54%
Postgraduate/doctorate				5.56%
Job situation				
Student, no paid work				0.33%
Student, paid work				4.58%
Employed (not student)				90.85%
Unemployed, looking for job				1.63%
Housewife/husband				0.98%
Cannot work				1.63%
Type of job				
Managerial/entrepreneur				11.76%
Professional/academic				23.86%
Employee/clerical				22.88%
Healthcare				10.46%
Sales/marketing/service				4.25%
Creative/artistic				1.63%
Other				20.59%
No answer				4.58%

The FSPPD was approved by the Ethics Committee of KU Leuven (reference OT 98/12 ZKA 2922). Written informed consent was obtained from all participants and from their parents when participants were underage.

### Measures

#### Personality traits

Big Five personality traits were assessed through the Hierarchical Personality Inventory for Children (HiPIC), a questionnaire specifically designed to assess personality differences from childhood onward and conceptually grounded in the Five Factor Model [27]. The HiPIC is composed of 144 items hierarchically organized in five higher‐order domains, namely extraversion, conscientiousness, emotional stability, benevolence and imagination. Although the first three dimensions are similar to the adult Big Five traits, benevolence and imagination reflect agreeableness and openness to experience in ways that are more observable and developmentally meaningful in younger age groups [28, 29]. Benevolence has slightly broader content than its Big Five counterpart, also capturing aspects of difficult temperament and antagonistic behavior. Imagination combines creativity and curiosity, main characteristics of Big Five's openness to experience, with intellect, the fifth trait from the lexical approach to personality [27, 30]. Factor analytic work with adolescent samples has demonstrated that HiPIC benevolence and imagination load on the same latent dimensions as adult agreeableness and openness to experience measured with the NEO Personality Inventory (NEO‐PI‐R) [9].

Parents rated the child's personality on a 5‐point Likert scale ranging from 1 (barely characteristic) to 5 (highly characteristic). We relied on averaged parent reports on child personality at T1 and on self‐reports onward. Parent‐ and self‐ratings showed high inter‐rater agreement [intraclass correlation coefficients (ICCs) = 0.76–0.89] [14], reflecting robust convergent validity across informants, consistent with cross‐informant findings reported in the same longitudinal sample [30]. The HiPIC has demonstrated structural stability into young adulthood [30, 31], with moderate to high rank‐order stability coefficients over time (r = 0.51–0.81). This makes it uniquely suited for longitudinal research, where maintaining the same instrument across different measurement occasions enhances comparability of measurements over time [32]. We, therefore, used it to assess personality across childhood, adolescence and emerging adulthood, ensuring measurement consistency. Cronbach's α ranged between 0.92 and 0.96 in mother and father's report, and between 0.86 and 0.92 in the child's report (Table 2).

**TABLE 2:** Descriptive characteristics of the HiPIC scales in childhood (T1, 6–9 years), adolescence (T2, 14–17 years) and emerging adulthood (T3, 20–23 years) and of the AUDIT in adulthood (T4, 29–32 years).

T1 (*n* = 306)‐childhood	M	SD	Min	Max	α	%
Extraversion	3.62	0.45	2.41	4.67	0.94	
Benevolence	3.47	0.44	1.64	4.90	0.96	
Conscientiousness	3.41	0.53	1.75	4.80	0.96	
Emotional stability	3.48	0.56	1.70	4.91	0.92	
Imagination	3.87	0.51	2.17	4.88	0.95	
T2 (*n* = 289)‐adolescence						
Extraversion	3.48	0.48	2.16	4.69	0.92	
Benevolence	3.52	0.40	2.20	4.88	0.89	
Conscientiousness	3.26	0.54	1.59	4.81	0.92	
Emotional stability	3.42	0.68	1.62	4.88	0.90	
Imagination	3.48	0.48	2.12	4.88	0.86	
T3 (*n* = 290)‐emerging adulthood						
Extraversion	3.43	0.53	1.94	4.81	0.92	
Benevolence	3.67	0.40	2.35	4.68	0.89	
Conscientiousness	3.53	0.54	1.84	4.78	0.91	
Emotional stability	3.24	0.76	1.31	5.00	0.92	
Imagination	3.68	0.44	2.50	4.83	0.86	
T4 (*n* = 306)						
AUDIT‐adulthood	5.39	4.37	0	31		
Low risk (0–7) (*n* = 241)						78.76
Medium risk (8–15) (*n* = 56)						18.3
High risk (16–19) and possible alcohol addiction (≥20) (*n* = 9)						2.94

Abbreviations: AUDIT, Alcohol Use Disorders Identification Test; HiPIC, Hierarchical Personality Inventory for Children.

#### Problematic alcohol use

Problematic alcohol use was assessed in adult individuals (29–32 years) through the Alcohol Use Disorders Identification Test (AUDIT) [33], a 10‐item screening tool developed by the WHO. Each item is in the form of a question, to which individuals answer with a 5‐point Likert scale, typically ranging from 0 (i.e. never) to 4 (i.e. daily or almost daily). The questionnaire includes items assessing alcohol use frequency, drinking behavior and alcohol‐related problems during the last year.

The AUDIT total score is a continuous measure that reflects the severity of problematic alcohol use, ranging between 0 and 40. In clinical and research settings, standard cut‐off scores are used to classify individuals into risk categories that represent progressively worsening levels of problematic alcohol use, as follows: low risk (0–7), medium level of problematic alcohol use (8–15), high level of problematic alcohol use (16–19) and possible alcohol addiction (≥20) [34]. It is worth noting that problematic alcohol use was assessed only in T4, because this variable was not included in previous waves of the FSPPD [25].

### Statistical analysis

All the analyses were performed using the statistical software R 4.4.0 [35]. First, socio‐demographic and descriptive statistics were reported. Second, preliminary data screening included an assessment of missing values, followed by the application of imputation procedures at each time point. Initially, participants with more than 70% missing data were excluded. Little's MCAR test was then performed to assess whether the missing data were completely random. Results indicated random missingness for all time points except T3 [χ^2^ (988) = 1195, *P* < 0.001] where non‐random patterns were detected. However, the overall proportion of missing data remained less than 1% across variables. Given this low rate of missingness, remaining values were handled through mean imputation. Third, the levels of personality traits at each wave were tested as possible necessary conditions for problematic alcohol use in adulthood through NCA [19]. NCA is an innovative method for testing necessary causality [19]. Establishing a necessity causal claim requires several conditions: (i) evidence of a statistically meaningful necessity relation between X and Y; (ii) temporal precedence (X occurs before Y); and (iii) a clear theoretical rationale for why X must be present for Y to occur [36]. Procedurally, NCA provides a scatterplot for each X–Y relationship (i.e. personality trait–problematic alcohol use) and identifies the area where all the empirically possible observations are, termed ‘scope’. The area where no observation is present is termed ‘ceiling zone’. If higher values of X are deemed as necessary for higher levels of Y, the ceiling zone is located in the upper left corner, whereas if lower levels of X are considered as necessary for higher levels of Y, the ceiling zone is located in the upper right corner. To quantify the ceiling zone, the primary approach is the ceiling envelopment‐full disposal hull (CE‐FDH) [19], which is a stepwise non‐decreasing linear function, which links all the highest values of the y‐axis for each value on the x‐axis, and it is recommended when conditions with few values are considered.

The presence of necessity in kind is evaluated by means of the NCA's effect size (*d*), which ranges from 0 to 1, and the threshold values considered for the significance of a necessary conditions are effect size *d* ≥ 0.10, and *P* ≤ 0.05 [17]. This effect size differs from Cohen's *d* as it is defined as the ratio between the ceiling zone and the scope, rather than representing a mean difference between groups. Its statistical significance is assessed through a permutation test (with canonically 10 000 permutations) [19]. In detail, it tests whether the observed *d* could plausibly arise by chance under a no‐relation null hypothesis by repeatedly permuting the X–Y pairings and recalculating *d*. Hence, the *P*‐value is the proportion of permuted datasets in which the simulated effect size equals or exceeds the observed. Therefore, *P* ≤ 0.05 suggests the empty space (necessity effect) is unlikely under randomness, providing evidence of necessity in the observed data [37]. The second step in NCA involves testing necessity in degree. This goal is achieved by means of the bottleneck analysis, which allows identifying which levels of the X variable are necessary for specific levels of the Y variable [19].

It is worth stressing that the NCA model is formulated as the inequality *Y* ≤ *f(X)*, where *f(X)* is the estimated ceiling line. Crucially, the specification contains no error term ε, because NCA does not aim to explain variation below the ceiling; in that sense, it specifies only a part of the data‐generating process (DGP) [36]. This differs from standard regression models of the form *Y* = *f(X)* + ε*(X)*, where the error term makes the relationship explicitly probabilistic and, therefore, part of a statistical model. As a result, the usual regression‐based notion of a ‘confounder’ is not directly applicable to NCA, because NCA does not model the conditional distribution of Y given X nor impose distributional assumptions on observations beneath the ceiling [36]. For this reason, the criterion of control for confounders to establish (necessity) causality does not apply to NCA [36].

In this study, we examined whether personality traits in childhood, adolescence and emerging adulthood are necessary conditions for problematic alcohol use severity in adulthood. The analytical plan was pre‐registered (https://osf.io/fxk8d). All datasets, analysis codes and a fully reproducible workflow are available (https://osf.io/7j8s2/overview?view_only=c55535991939401f99433fc15ff13499). All analyses were conducted using the NCA package version 4.0.2 in R 4.5.1 in RStudio Version 2025.5.1.513.

## RESULTS

Descriptive statistics are reported in Table 2.

### Necessity in kind

**FIGURE 1:**
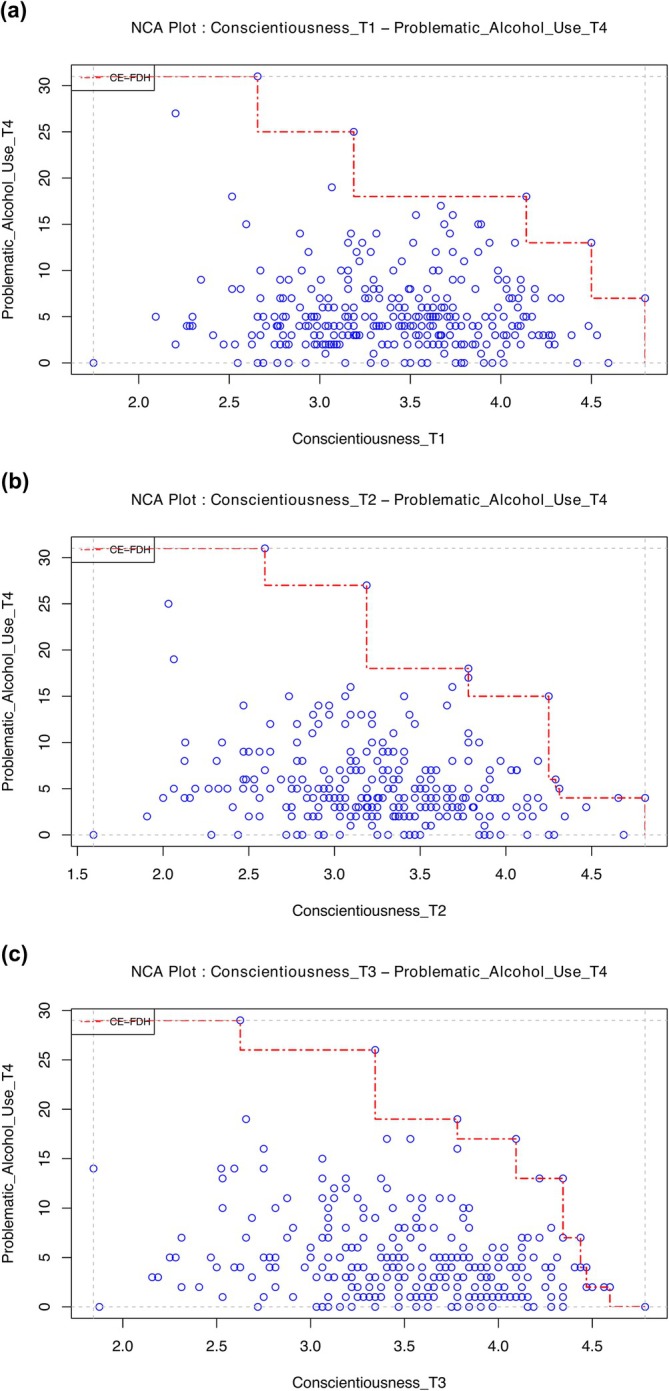
Necessary condition analysis (NCA), illustrating the necessity relationship between problematic alcohol use (Y) and conscientiousness at T1 (childhood), T2 (adolescence) and T3 (emerging adulthood).

In T1 (childhood) and T3 (emerging adulthood), conscientiousness was a statistically significant necessary condition for the presence of problematic alcohol use at T4 (adulthood), such that lower levels of conscientiousness were necessary for higher levels of problematic alcohol use (respectively, *d* = 0.31, large effect, *P* = 0.050 and *d* = 0.30, large effect, *P* = 0.023) (Figure 1). Although the magnitude of the effect was the same in T2 (adolescence; *d* = 0.33, large effect), no full statistical significance was reached (*P* = 0.052). Given the remarkable consistency of the result through the years, we proceeded with the analysis for all three waves. No other personality traits were statistically significant necessary conditions (i.e. *P* > 0.05) for problematic alcohol use at any considered time. All the NCA results are reported in Table 3.

**TABLE 3:** Effect size and significance of the Big Five personality traits in childhood (T1, 6–9 years), adolescence (T2, 14–17 years) and emerging adulthood (T3, 20–23 years) as necessary conditions for problematic alcohol use in adulthood (T4, 29–32 years).

	T1‐childhood		T2‐adolescence		T3‐emerging adulthood	
	*d*	*P*	*d*	*P*	*d*	*P*
Extraversion	0.28	0.28	0.27	0.37	0.19	0.73
Benevolence	0.24	0.52	0.25	0.72	0.23	0.32
Conscientiousness	0.31	0.05	0.33	0.052	0.30	0.02
Emotional Stability	0.23	0.39	0.1	0.86	0.22	0.43
Imagination	0.23	0.88	0.28	0.25	0	1

*Note*: The effect size *d* is based on ceiling envelopment‐full disposal hull.

### Necessity in degree

Given the limited number of participants that scored in the possible alcohol addiction range (AUDIT ≥20), this category was merged with the adjacent high‐risk group. Accordingly, the bottleneck analysis reports only two levels of problematic alcohol use, namely medium (AUDIT 8–15) and high (AUDIT ≥16). The number of participants across the cut–off‐based levels is reported in Table 2.

The bottleneck analysis revealed that to possibly show AUDIT scores corresponding to medium levels of problematic alcohol use (8–15) in adulthood, conscientiousness scores in childhood should not exceed 4.5, 4.25 in adolescence and 4.34 in emerging adulthood. In other words, a person could maybe show medium levels of problematic alcohol consumption in adulthood, if and only if they reported a conscientiousness score of 4.5 or less in childhood. Furthermore, to possibly show AUDIT scores corresponding to high levels of problematic alcohol use (≥16) in adulthood, conscientiousness scores in childhood should not exceed 4.14, 3.78 in adolescence and 4.09 in emerging adulthood (Table 4). It is remarkable that the conscientiousness levels required for each AUDIT level were strongly stable across the three waves. Bottleneck analyses by AUDIT score indicated that conscientiousness provides sufficient granularity to effectively constrain differences in problematic alcohol use (Table S1). Bottleneck analyses stratified by gender are also presented (Table S2). These results indicate that male and female participants exhibit comparable required levels of conscientiousness, although females descriptively report slightly lower levels.

**TABLE 4:** Bottleneck table for conscientiousness as necessary condition for problematic alcohol use.

AUDIT T4 (29–32 years)	Conscientiousness T1 (7–9 years)	Conscientiousness T2 (14–17 years)	Conscientiousness T3 (20–23 years)
8–15 (medium risk)	4.5	4.25	4.34
≥16 (high risk)	4.14	3.78	4.09

Abbreviation: AUDIT, Alcohol Use Disorders Identification Test.

Importantly, the bottleneck analysis allowed differentiating individuals who showed the necessary condition for reporting the different levels of problematic alcohol use in adulthood from the ones who did not (Table 5). Focusing on the most severe level (high, AUDIT ≥16): during childhood, 93.46% of participants showed the required conscientiousness level for potentially showing high levels of problematic alcohol use in adulthood. Of this group, only 2.82% reported high levels of problematic alcohol use. In adolescence, 80.97% of participants showed the required conscientiousness level for potentially showing high levels of problematic alcohol use in adulthood. Of this group, only 2.91% reported high levels of problematic alcohol use. In emerging adulthood, 84.14% of participants showed the required conscientiousness level for potentially reporting high levels of problematic alcohol use in adulthood. Of this group, only 3.35% showed high levels of problematic alcohol use. From this analysis, it follows that 6.54% of the sample in childhood, 19.03% in adolescence and 15.86% in emerging adulthood were virtually immune to showing high levels of problematic alcohol use.

**TABLE 5:** Bottleneck levels for conscientiousness as a necessary condition for different levels of problematic alcohol use, percentage of participants who met the necessary condition, percentage of participants with problematic alcohol use of those that met the necessary condition and percentage of participants who did not meet the necessary condition.

AUDIT	Necessary level	% with necessary condition	% with problematic alcohol use of those with necessary condition	% without necessary condition
Childhood				
8–15 (medium level)	4.5	99.02	21.45	0.98
≥16 (high level)	4.141	93.46	2.82	6.54
Adolescence				
8–15 (medium level)	4.25	97.23	22.03	2.77
≥16 (high level)	3.781	80.97	2.9	19.03
Emerging adulthood				
8–15 (medium level)	4.344	96.55	21.17	3.45
≥16 (high level)	4.094	84.14	3.35	15.86

Abbreviation: AUDIT, Alcohol Use Disorders Identification Test.

### Sensitivity analysis

We assessed robustness by increasing the precision of our estimates and conducting a sensitivity analysis of extreme values. First, we re‐ran the analyses using 50 000 permutations. Conscientiousness emerged as a fully significant necessary condition in childhood (*d* = 0.31, large effect, *P* = 0.050), adolescence (*d* = 0.33, large effect, *P* = 0.049) and emerging adulthood (*d* = 0.30, large effect, *P* = 0.025). Second, we removed cases that altered the estimated necessity effect by 10% or more. The results largely aligned with the primary findings: conscientiousness remained a significant necessary condition in childhood (*d* = 0.38, large effect, *P* < 0.04) and was clearly significant in adolescence (*d* = 0.44, large effect, *P* < 0.001). In emerging adulthood, conscientiousness was no longer significant (*P* < 0.17), although the effect size remained of moderate magnitude (*d* = 0.11). No other personality trait emerged as a statistically significant necessary condition.

## DISCUSSION

Alcohol consumption is a widespread behavior, and a substantial body of literature has identified personality features as risk factors for alcohol consumption patterns, including frequency and problematic use [9, 38, 39]. However, to date, no study has investigated whether a specific pattern of personality traits is necessary for the presence of such a health‐risk behavior in adulthood. In the current study, we used the NCA [19] approach to test whether personality traits in childhood, adolescence and emerging adulthood act as necessary conditions for problematic alcohol use in adulthood, and if so, which ones and to what degree.

Our findings indicate that conscientiousness is the only personality trait among those tested that consistently serves as a necessary condition, from childhood through emerging adulthood, for the presence of problematic alcohol use in adulthood. Conscientiousness comprises multiple inter‐related facets, including concentration (e.g. ‘I can keep my thoughts in the same place for a long time’), perseverance (e.g. ‘I persevere until the goal is reached’), achievement motivation (e.g. ‘I am committed to something with all my heart’) and orderliness [e.g. ‘I take care of (my) own possessions’] [27]. Lower conscientiousness is typically associated with less consistent planning and self‐regulation and a stronger tendency for immediate rewards [27]. More broadly, low conscientiousness has been conceptualized as greater vulnerability to self‐regulatory difficulties [40], which may contribute over time to the emergence of health‐risk behaviors [16].

Problematic alcohol use is often seen as the result of a difficulty of self‐regulation, involving impulsivity, impaired inhibitory control and excessive reliance on automatic processing [41]. The dual process model [42] describes behavior as shaped by the interaction between a fast, impulsive system and a slower, reflective one. Reflective control depends on cognitive resources, time and motivation to engage top‐down processes that guide attention and inhibit automatic responses. The reinforcement/reprocessing model (R^3^) [42] suggests that when these functions are not activated—because of reduced motivation, limited cognitive capacity or weak reinforcement history—impulsive processing takes over. Therefore, individuals low in conscientiousness may be especially vulnerable, because they often show reduced executive control and struggle to delay gratification, limiting their ability to engage reflective processing in high‐risk situations. In this sense, low conscientiousness may be regarded as a theoretically grounded candidate as a necessary condition for problematic alcohol use. Conversely, high conscientiousness enhances reflective capacity, offering protection against less adaptive responses. This enhanced regulatory capacity may serve as an immunity 1 factor for those who carry it, nullifying their vulnerability to problematic alcohol consumption.

Identifying conscientiousness as a necessary condition for problematic alcohol use underscores the importance of understanding how this trait itself develops and is shaped by environmental influences. Personality traits unfold across development through ongoing interactions between dispositional tendencies and contextual experiences, including early‐life adversities [44, 45]. In this respect, facets of low conscientiousness (e.g. reduced perseverance or attentional control) may partly reflect self‐regulatory adaptations to prolonged stress or adverse relational environments. Moreover, longitudinal evidence shows that alcohol use prospectively predicts small but consistent decreases in conscientiousness over time [46]. Future research should examine which conditions are necessary for specific levels of conscientiousness and whether additional factors also emerge as necessary conditions for later problematic alcohol use.

A clarification regarding our results is warranted. NCA adopts a deterministic or typicality perspective rather than a probabilistic one [17]. This implies that individuals who do not meet a necessary condition are considered virtually immune to a given outcome, rather than merely at lower risk of experiencing it [21]. However, necessity is not expected to hold universally, rather, it applies specifically within a defined theoretical domain—that is, the universe of instances of a focal unit of a theory, proposition or hypothesis for which the theory, proposition or hypothesis is intended to hold [36]. In the present study, we focused exclusively on individuals from the Flemish community, although cultural factors can substantially influence both personality [47] and alcohol consumption [48]. Therefore, future research is needed to replicate and extend our findings and to examine their generalizability across different cultural contexts. Furthermore, NCA studies—like more traditional methodological approaches—are limited to the specific sample and the particulars of the study design. Consequently, our findings cannot be generalized to infer vulnerability for problematic alcohol use in individuals outside the assessed age range (29–32 years).

Our results and, more broadly, the NCA method yield important theoretical and clinical implications [21]. First, our analyses suggest that, across the lifespan, it is possible to identify characteristics that may help distinguish individuals at risk of developing problematic alcohol use in adulthood. This indicates that conscientiousness, as a necessary condition, is a somewhat stable precondition, whose constraining/enabling role persists for a very long part of the lifespan. Second, our results can be integrated in two distinct but not mutually exclusive approaches, to further develop theoretical models. One perspective is that sufficiency and necessity relationships may be incorporated in a combined theory, namely ‘embedded necessity theory’ [49]. For example, the Gladwin and collaborators’ [42] model could be integrated with conscientiousness as a necessary condition for the successful operation of the reflective system. Embedding such a necessity constraint may sharpen predictions regarding who is at risk and under what conditions self‐regulation does not perform optimally. However, these types of complex embedded models, although highly informative, would most probably be difficult to test and potentially falsify [50]. Another perspective—a simpler and more testable one—would be building pure necessity theories, composed solely of necessity relationships [49]. In this case, the theory would state which conditions are necessary for a negative outcome, meaning that the absence of just one of those would not allow the outcome. By drawing on the most up‐to‐date theory‐building methods, it should be possible to develop pure necessity theories that yield highly testable (and falsifiable) predictions.

Important clinical implications can be derived too. First, pending future replications and extensions, our study suggests that screening for conscientiousness already in adolescence, and perhaps even in childhood, could serve as an indicator of vulnerability, whereas individuals with above‐average levels could be defined as virtually immune from showing problematic alcohol use later in life. This would allow for the implementation of selective preventive interventions [21, 22]. Second, NCA's deterministic logic is among its most thought‐provoking features, with important consequences. After firmly establishing in‐kind and in‐degree necessity, as well as the precise bottleneck levels, across multiple samples within the same theoretical domain [19, 21], NCA‐derived group‐level evidence could be leveraged to inform individual‐level assessment and guide preventive and clinical indications [20, 21]. Third, targeting conscientiousness might be a potential way to hinder problematic alcohol use. A recent systematic review has discussed several conscientiousness interventions, finding out that increasing the trait is possible and that the results are mostly durable [51]. Following the necessary condition logic, enhancing the trait level just above the necessary threshold may block the presence of problematic alcohol use [20, 21].

Several limitations should be considered. First, these findings require replication in independent samples, ideally more diverse in country, gender and ethnicity. Second, the study relied on questionnaire‐based reports, and future work should incorporate clinical interviews, particularly for problematic alcohol use, to strengthen validity. Third, problematic alcohol use was assessed only in adulthood (covering the prior 12 months), preventing analyses of developmental trajectories and whether conscientiousness is necessary for onset, maintenance or both [20]. Fourth, few participants were classifiable as potentially carrying an alcohol addiction, which may limit precision for the highest‐severity bottlenecks. Fifth, after removing potential influential cases, conscientiousness was no longer statistically significant in emerging adulthood, suggesting some instability in the pattern of necessary conditions at this developmental stage. Nevertheless, across the lifespan, the magnitude of the association for conscientiousness remained moderate to large. Future studies should adopt a more fine‐grained temporal resolution to better capture the stability and dynamics of necessary conditions for problematic alcohol use.

In conclusion, this study provides valuable insights into the investigation of necessary conditions in both the personality and problematic alcohol use domains. The longitudinal evidence indicates that, although preliminary, low‐to‐moderate conscientiousness may constitute a bottleneck for the subsequent development of problematic alcohol use. We maintain that adopting a necessary condition perspective in addiction research may offer novel and illuminating avenues for enhancing the understanding, treatment and prevention of health‐risk behaviors.

## AUTHOR CONTRIBUTIONS

**Angela Giugovaz:** Investigation; writing—original draft; visualization; formal analysis; writing—review and editing; data curation. **Peter Prinzie:** Writing—review and editing; resources; data curation; funding acquisition. **Miranda C. Lutz:** Writing—review and editing; supervision. **Ingmar H. A. Franken:** Writing—review and editing; supervision. **Igor Marchetti:** Conceptualization; methodology; funding acquisition; supervision; project administration; writing—review and editing.

## DECLARATION OF INTERESTS

None.

## Supporting information


**Table S1.** Bottleneck table for Conscientiousness as a necessary condition for Problematic Alcohol Use by outcome level.
**Table S2.** Bottleneck table for Conscientiousness as necessary condition for Problematic Alcohol Use by gender. Note. We thank the editor and reviewers for suggesting this additional set of analyses.

## Data Availability

All datasets, analysis codes, and a fully reproducible workflow are available (https://osf.io/7j8s2/overview?view_only=c55535991939401f99433fc15ff13499).

## Footnotes

^1^
Because the necessary condition analysis (NCA) is grounded in a deterministic framework, we propose that an unmet necessary condition can be conceptualized as an immunity factor—a condition whose absence effectively prevents the onset of a disorder in virtually all cases [22]. This notion of an immunity factor stands in contrast to traditional protective factors, which are typically defined as probabilistic sufficient conditions—variables statistically associated with a reduced likelihood of developing a disorder [43].
