# Supplementary material for: Which personality traits are necessary conditions for problematic alcohol use? Insights from a 23‐year longitudinal study
Source: Addiction. 2026 Apr 7;121(7):1824–35. doi: 10.1111/add.70417 (PMC13291086; doi:10.1111/add.70417)
Supplement: Supplementary file 1 — Table S1. Bottleneck table for Conscientiousness as a necessary condition for Problematic Alcohol Use by outcome level. Table S2. Bottleneck table for Conscientiousness as necessary condition for Problematic Alcohol Use by gender. Note. We thank the editor and reviewers for suggesting this additional set of analyses. [file ADD-121-1824-s001.docx]

**Supplementary Material**

**Table S1.** *Bottleneck table for Conscientiousness as a necessary condition for Problematic Alcohol Use by outcome level.*

| AUDIT T4  (29-32 years) | Conscientiousness T1  (7-9 years) | Conscientiousness T2  (14-17 years) | Conscientiousness T3  (20- 23 years) |
| --- | --- | --- | --- |
| 0 | 4.797 | 4.812 | 4.781 |
| 1 | 4.797 | 4.812 | 4.594 |
| 2 | 4.797 | 4.812 | 4.594 |
| 3 | 4.797 | 4.812 | 4.469 |
| 4 | 4.797 | 4.812 | 4.469 |
| 5 | 4.797 | 4.312 | 4.438 |
| 6 | 4.797 | 4.29 | 4.438 |
| 7 | 4.797 | 4.25 | 4.438 |
| 8 | 4.5 | 4.25 | 4.344 |
| 9 | 4.5 | 4.25 | 4.344 |
| 10 | 4.5 | 4.25 | 4.344 |
| 11 | 4.5 | 4.25 | 4.344 |
| 12 | 4.5 | 4.25 | 4.344 |
| 13 | 4.5 | 4.25 | 4.344 |
| 14 | 4.141 | 4.25 | 4.094 |
| 15 | 4.141 | 4.25 | 4.094 |
| 16 | 4.141 | 3.781 | 4.094 |
| 17 | 4.141 | 3.781 | 4.094 |
| 18 | 4.141 | 3.781 | 3.781 |
| 19 | 3.188 | 3.188 | 3.781 |
| 20 | 3.188 | 3.188 | 3.344 |
| 21 | 3.188 | 3.188 | 3.344 |
| 22 | 3.188 | 3.188 | 3.344 |
| 23 | 3.188 | 3.188 | 3.344 |
| 24 | 3.188 | 3.188 | 3.344 |
| 25 | 3.188 | 3.188 | 3.344 |
| 26 | 2.656 | 3.188 | 3.344 |
| 27 | 2.656 | 3.188 | 2.625 |
| 28 | 2.656 | 2.594 | 2.625 |
| 29 | 2.656 | 2.594 | 2.625 |
| 30 | 2.656 | 2.594 | NA |

**Table S2.** *Bottleneck table for Conscientiousness as necessary condition for Problematic Alcohol Use by gender.*

| AUDIT T4  (29-32 years) | Conscientiousness T1 (7-9 years) | | Conscientiousness T2 (14-17 years) | | Conscientiousness T3 (20- 23 years) | |
| --- | --- | --- | --- | --- | --- | --- |
|  | Male | Female | Male | Female | Male | Female |
| 0 | 4.5 | 4.797 | 4.812 | 4.688 | 4.594 | 4.781 |
| 8 | 4.5 | 4.188 | 4.25 | 3.781 | 4.344 | 3.844 |
| 16 | 3.734 | 4.141 | 3.781 | 3.781 | 4.094 | 3.781 |
| *Note.* We thank the editor and reviewers for suggesting this additional set of analyses | | | | | | |
